# In What Ways Does Health Related Stigma Affect Sustainable Employment and Well-Being at Work? A Systematic Review

**DOI:** 10.1007/s10926-021-09998-z

**Published:** 2021-09-06

**Authors:** I. E. van Beukering, S. J. C. Smits, K. M. E. Janssens, R. I. Bogaers, M. C. W. Joosen, M. Bakker, J. van Weeghel, E. P. M. Brouwers

**Affiliations:** 1 Tranzo, Scientific Center for Care and Wellbeing, Tilburg School of Social and Behavioral Sciences, Tilburg University, Tilburg, The Netherlands; 2 Inspectorate SZW, Den Haag, The Netherlands; 3 P.O. Box 90513, 5000 LE Tilburg, The Netherlands; 4 Summa College, Eindhoven, The Netherlands; 5 Brain Research and Innovation Centre, Ministry of Defense, Den Haag, The Netherlands; 6 Department of Methodology and Statistics, Tilburg School of Social and Behavioral Sciences, Tilburg University, Tilburg, The Netherlands; 7 Phrenos Center of Expertise, Utrecht, The Netherlands

**Keywords:** Stigma, Discrimination, Disability, Well-being, Employment

## Abstract

**Purpose:**

Studies are increasingly showing that health related stigma is a barrier to employment, but it is not known how. The aim of this systematic review is to identify, appraise and analyse studies that have directly or indirectly addressed ways in which stigma affects sustainable employment and well-being at work of people with disabilities.

**Methods:**

Using a multiphase screening process, this review is based on a comprehensive literature search (2000–2019) carried out in six electronic databases: Embase, Web of Science, Medline Ovid, Cochrane CENTRAL, PsycINFO and Google Scholar.

**Results:**

7.263 publications were identified; 96 studies were found eligible to be included in the review. 72% of the studies were conducted in North America or Europe. Few studies directly assessed how stigma affects the employment of people with disabilities. Most studies highlighted that attitudes and behaviour of employers formed a barrier to employment, as well as anticipated stigma and self-stigma in people with health problems. However, the findings also showed that the attitudes and behaviour of co-workers, health care professionals, reintegration professionals, customers, and family and friends could act as a barrier to employment although these influences are under-researched. Although many similarities were seen in the relevant findings of studies about both physical and mental disabilities, several nuances were found.

**Conclusion:**

Stigma hampers sustainable employment and well-being in multiple ways. Whereas the number of publications on this topic is rapidly increasing, the roles of health care professionals, reintegration professionals, co-workers, customers, and family and friends particularly warrant more attention.

**Supplementary Information:**

The online version contains supplementary material available at 10.1007/s10926-021-09998-z.

## Introduction

Previous research has shown that people with common and severe mental disorders are three to seven times more likely to be unemployed compared to people with no disorders [1]. In addition, people with physical disabilities are twice as often unemployed compared to their nondisabled counterparts [2]. It is important that people with disabilities can participate in the labour market as people with mental and physical disorders could benefit from the positive aspects of employment, such as structure time, routine, and social contact [3, 4]. In addition, studies have shown that unemployment has a negative impact on health, such as psychological distress and depression [5]. Furthermore, unemployment has been associated with social problems, such as poverty and increased costs for society [6, 7].

Health related stigma has been suggested to be a major problem for people with disabilities when it comes to sustainable employment (finding and/or keeping work) and to well-being at work [8]. Stigma is a process of negatively labelling, condemning and excluding a certain group of people from society [9]. According to Thornicroft et al., stigma arises as a result of inadequate or biased knowledge about, in the case of the present study, mental and physical disabilities [10]. Stigma can lead to discrimination. Discrimination is the behavioural consequence of stigma, which leads to the disadvantage of people who are being stigmatized [11]. Unlike stigma, discrimination is prosecutable, enabling stakeholders in many countries to act in accordance to anti-discrimination policies and laws [10].

In the literature on stigma, various factors have been suggested through which stigma can lead to unemployment and other adverse occupational outcomes although most articles do not directly focus on how stigma affects sustainable employment and well-being at work. Rigorous systematic reviews in this area are lacking [8]. One of the suggested factors is that employers and other stakeholders in the work environment often hold negative attitudes towards people with disabilities, which decreases the chances of these people to be hired or supported at work [12–16]. Many people with health problems experience a disclosure dilemma [17–20]. Whereas disclosure of a disability could prevent job loss as it could lead to work adjustments, disclosure also causes job loss due to stigma [18]. Health related stigma has also been reported to be a barrier to seeking healthcare. Untreated and worsened health conditions could subsequently lead to unemployment [8, 21–23]. Finally, anticipated stigma, self-stigma and the ‘Why Try’ effect could lead to insufficient motivation and effort to keep or find employment [9, 24]. Anticipated stigma means that people with health problems expect to be stigmatized [8]. Self-stigma can lead to questioning whether it is worth pursuing personal goals, such as applying for a job. This so-called ‘Why Try’ effect can result in an insufficient effort to find or keep a job, which increases the risk of unemployment [9]. The kind and degree of stigmatization may vary depending on the type of disability, visibility of the disability, and symptom severity [18].

Despite of the fact that the number of scientific publications on workplace stigma seems to be increasing and publications show that stigma in the work context is a considerable and complex problem for people with mental and physical disabilities, knowledge about how health related stigma acts as a barrier to sustainable employment and well-being at work is scarce. Moreover, to the best of our knowledge, systematic reviews on how health related stigma affects sustainable employment and well-being at work have not been performed yet. Therefore, the aim of this systematic review is to identify, appraise and analyse studies that have directly or indirectly addressed ways in which health related stigma affects sustainable employment and well-being at work of people with disabilities.

## Methods

### Search Strategy

The databases of Embase, Medline Ovid, Web of science Core Collection, Cochrane CENTRAL, PsycINFO and Google scholar were searched for articles published in English between January 2000 and July 2019. Articles in other languages than English were excluded because authors were not fluent in other foreign languages than English and the search was expected to already yield a large number of papers. Search strategies were developed and refined by translating our research question according to the PICO method [25] This resulted in three relevant groups: (1) Patient/population: physical or mental disabilities, (2) Intervention/exposure: stigma or discrimination, and (3) Outcome: well-being at work or sustainable employment (finding and/or keeping work). Including a control component was not relevant given our research question. For each of the groups we included terms and/or synonyms that were used as subject headings and/or text words (see Online Appendix 1). Articles were also identified by screening studies from reference lists of other relevant articles and references recommended by colleagues within the field. This review was designed and conducted according to the PRISMA statement for reporting systematic reviews [26].

### Selection Criteria

To be included, the title or abstract of the studies had to meet the following inclusion criteria: (1) related to physical and/or mental disabilities, (2) related to stigma and/or discrimination, and (3) related to work. Articles that fulfilled all three of the criteria or that the reviewers were uncertain about proceeded to the next selection phase. In the next phase of full-text screening, studies were included if they met the following eligibility criteria: (1) full text with abstract, (2) original peer reviewed journal article (no letters to the editor, editorials, comments, or reports), (3) based on empirical research: qualitative, quantitative and mixed methods studies (no reviews), (4) addresses health related stigma, (5) relationship with regular paid work (no sheltered employment and volunteer work), and (6) discusses ways in which health related stigma affects sustainable employment and/or well-being at work. Disability was regarded as a physical or mental condition; the variety of conditions searched for in this review can be seen in Online Appendix 1. This review did not distinguish between having a medical diagnosis and having a disability, although this does not have to be exactly the same. This review is a Mixed Studies Review (MSR), i.e., it includes qualitative, quantitative and mixed methods studies. By integrating qualitative, quantitative and mixed methods studies, this study enhanced its utility and impact because it enables combining quantitative knowledge on estimates with a qualitative understanding of the matter [27].

All studies were independently evaluated. In the first step, the title and abstract were reviewed by four reviewers (IB, SS, RB and KJ) using the inclusion criteria. In the second step the full-text articles were assessed by the same reviewers using the eligibility criteria. Per screening round each study was reviewed by two reviewers separately. Differences between the findings were discussed by the reviewers until consensus was reached.

### Quality Appraisal

The quality appraisal was conducted using the scoring system for MSR’s of Pluye, Gagnon, Griffiths and Johnson-Lafleur to concomitantly appraise the methodological quality of qualitative, quantitative and mixed methods studies [28]. This scoring system contains 15 quality criteria in total. The criteria differ per type of study. For each of the relevant criteria (see Online Appendix 2), the presence/absence may be scored 1 or 0 respectively. Articles that scored less than 50% on the quality criteria were removed from the data selection. Two reviewers (IB and SS) independently assessed the studies. A random selection of 10% of the studies was screened by two other reviewers (RB and KJ). There were no discrepancies among the researchers.

### Data Extraction and Analysis

The content of the included studies was extracted with regard to the following topics: country where the study was conducted, study population, sample size, target health problem, objectives and study design (see Table 1, Online Appendix 3), and relevant main results and conclusions for the purpose of this study. This was done by two reviewers (IB and SS). Four other reviewers (RB, KJ, EB and MJ) checked the data extraction. Differences were discussed by the reviewers until consensus was reached.

All included studies were synthesized using thematic content analysis. First, a thematic subdivision was made. The themes were based on the known stigma barriers from the existing literature: (1) attitudes and behaviour of employers, (2) anticipated stigma, self-stigma and the ‘Why Try’ effect by people with disabilities themselves [8]. Second, if findings could not be classified under these two categories, a new category was added. Third, within the categories, a distinction was made between findings that reported on attitude or behaviour. Fourth, subcategories were defined within the categories.

## Results

### Selected Studies

**Fig. 1:**
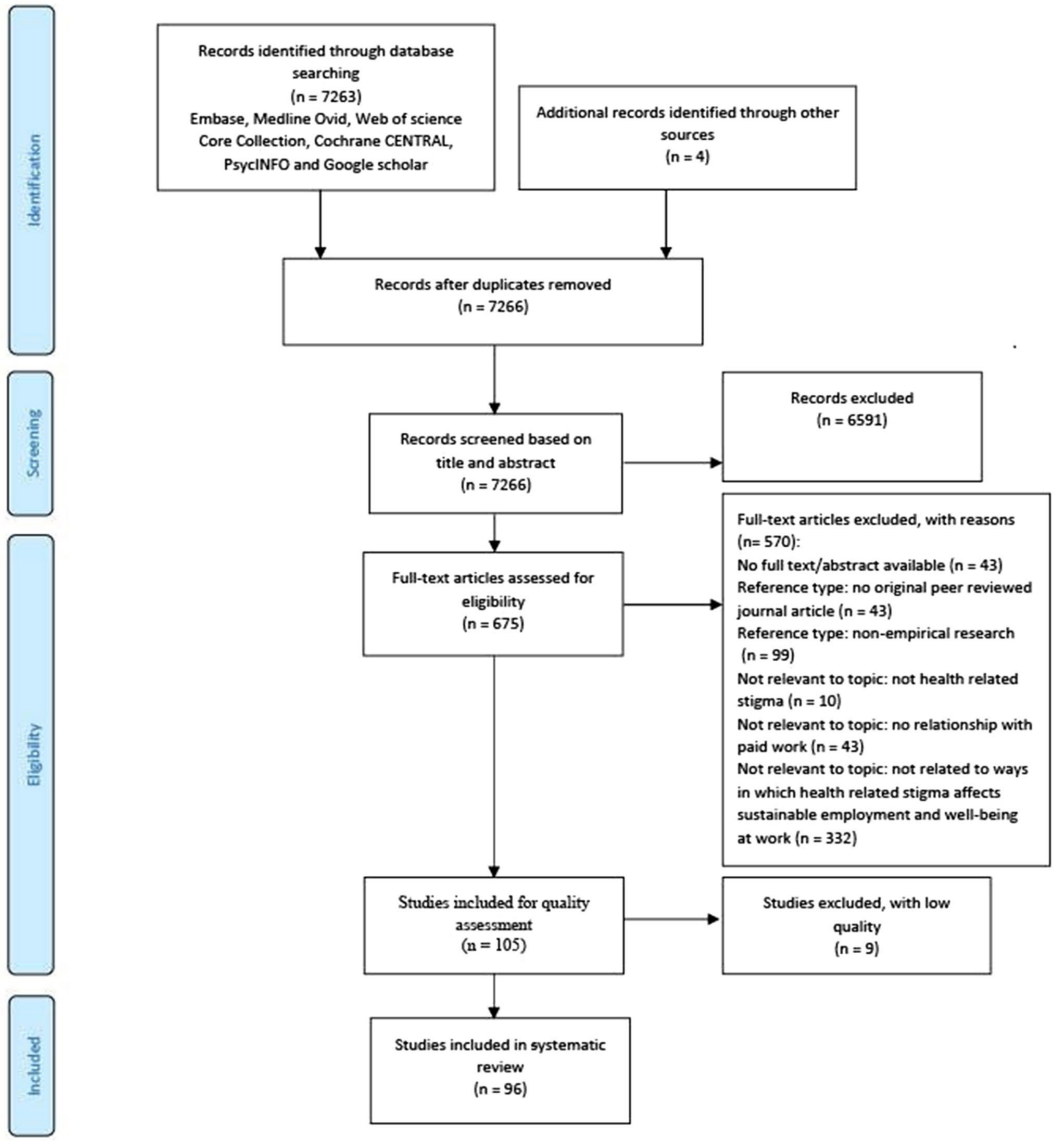
Flowchart of search

A PRISMA flow diagram of the study selection process is shown in Fig. 1. After excluding duplicates, 7266 references were retrieved from the databases and assessed based on title and abstract. The full texts of 675 potentially eligible articles were then examined, of which 105 articles met the inclusion criteria, nine of which did not meet the quality criteria, resulting in 96 articles included in this review.

### General Findings

**Fig. 2:**
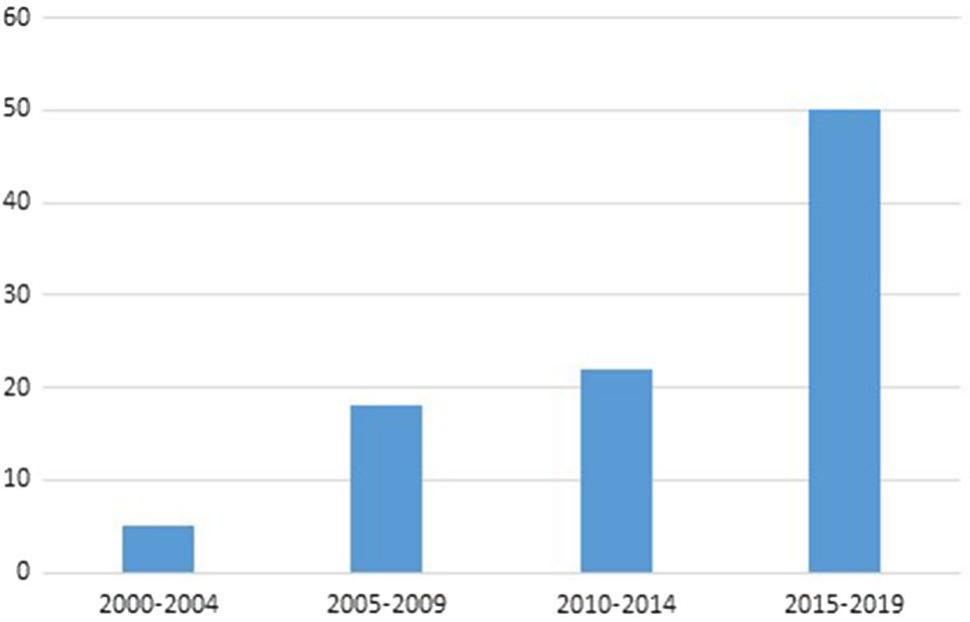
Selected studies by year of publication (N = 96)

Figure 2 shows that more than half of the selected articles were published between 2015 and 2019. The higher number of recently published studies was seen for both mental disability and physical disability studies.

**Fig. 3:**
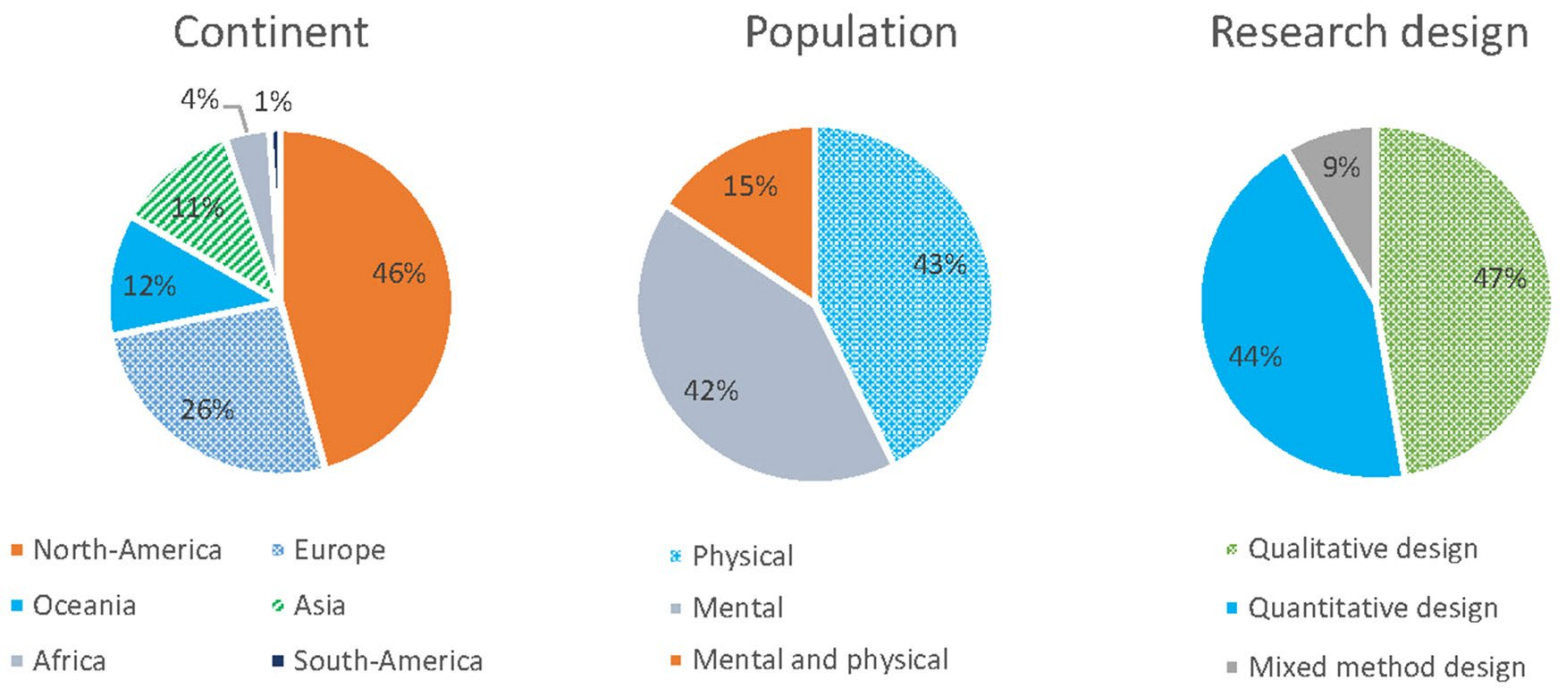
Continent, targeted population, and research design of selected studies (N = 96)

Studies from 27 different countries were included. Of the 96 studies, 72% of the studies were conducted in North America of Europe (see Fig. 3). In Western countries, research mainly focused on stigma related to mental disabilities. Stigma research in non-Western countries was mainly about stigma related to physical health problems, in particular communicable diseases such as HIV and Hepatitis.

Of the 46 qualitative studies, 38 studies conducted interviews or focus groups, four studies had an experimental design, and three studies used surveys with open-ended questions that were analysed using qualitative methods. There was one phenomenological case study. Of the 41 quantitative studies included in this review, 38 studies used questionnaires. There were two experimental studies. One of them used fake job applications aimed at real job vacancies to explore differences in expressions of employer interest. Another experimental study used vignettes to let employers make hiring decisions where the disability type and extent of disclosure were manipulated. There was one constructivist grounded theory methodology. All of the nine studies with a mixed method design used surveys and interviews or focus groups.

### Health Related Stigma, Sustainable Employment, and Well-Being at Work

The results of the findings are presented in Table 1. In Table 2, some example quotes from some of the qualitative papers can be found, which illustrate the findings presented in Table 1.

**Table 1:** Ways in which health related stigma affected sustainable employment and well-being at work of people with disabilities

Main outcomes	Attitude/behaviour	Evidence from systematic review
Health related stigma by *employers* is a barrier for people with disabilities to sustainable employment and well-being at work	Stigmatizing attitudes	Negative attitude was caused by a lack of knowledge of disabilities [13, 29–35]
		Employers perceived workers with disabilities as incompetent [15, 30, 32, 35–40]
		Employers believed worker with disabilities are dangerous [32, 38, 41–44]
	Behaviour affecting sustainable employment	Hiring discrimination made it challenging to find work because of the reluctance of employers to hire people with disabilities[17, 20, 30, 33, 37, 39, 41, 43, 45–63]
		Advancement-related discrimination was a barrier to maintaining work because of a lack of recognition or because they could not cope with the discrimination which made them quit their job, it could also lead to wrongful dismissal [33, 34, 36, 37, 47, 50, 53, 54, 59, 64–75]
		Refusing reasonable workplace accommodations made it difficult to maintain a job because a lack of accommodations made it hard to meet the expectations of their employer [35, 37, 47, 59, 60]
	Behaviour affecting well-being at work	Bullying, harassment and social discrimination negatively affected the well-being at work by feeling socially excluded and losing the motivation to perform the job [19, 35, 47, 48, 59, 65, 70, 76, 77]
Health related stigma by *co-workers* is a barrier for people with disabilities to sustainable employment and well-being at work	Stigmatizing attitudes	Negative attitude caused by a lack of knowledge of disabilities [13, 60]
		Co-workers perceived people with disabilities as incompetent [37, 65, 78–81]
		Co-workers challenged the fairness of preferential treatment [15, 37, 78]
	Behaviour affecting sustainable employment	Co-workers had lower intentions of working with people with disabilities, this could lead to increased tension and strained work relationships, which was a barrier in maintaining work [37, 60, 63, 65, 78, 80]
	Behaviour affecting sustainable employment and well-being at work	Harassment and bullying negatively affected someone’s well-being at work and chance to maintain a job by making them upset and lowering their self-esteem, this could lead them to resign [37, 65, 66, 70, 74, 75, 81–84]
		Social exclusion led to a diminished well-being at work and was barrier in maintaining work, it could lead to strained relationships and a loss of motivation to perform a job [37, 48, 60, 66, 77, 85–90]
Health related stigma by others: *health care professionals, reintegration professionals, customers, family and friends* is a barrier to sustainable employment	Behaviour affecting sustainable employment	(Mental) health professionals discouraged people with disabilities in finding and maintaining work [15, 91–94]
		Reintegration professionals thought clients were unable to handle the position and therefore they were less inclined to refer them to a job [62]
		Family and friends feared the stress of work and giving up benefits and discourage people with disabilities to get a job [91]
		Stigma by customers could result in negative evaluations of a disabled employee which could harm the chance of maintaining a job [56]
For *people with disabilities* anticipated stigma, self-stigma and ‘why try’ –effect, and the disclosure dilemma is a barrier to sustainable employment and well-being at work	Behaviour affecting sustainable employment	Anticipated stigma discouraged people with disabilities from pursuing and maintaining work [19, 31, 33, 48, 53, 61, 73, 77, 83, 92, 95–104]
		Self-stigma and ‘why try’-effect led to insufficient motivation and effort to find or maintain work [15, 22, 24, 39, 62, 66, 69, 84, 91, 92, 96, 103–115]
	Behaviour affecting sustainable employment and well-being at work	Disclosure dilemma negatively affected sustainable employment due to discrimination and because of the stress involving the decision whether to disclose or not to disclose a disability [19, 20, 60, 64, 72, 82, 116, 117]

**Table 2:** A selection of quotes from qualitative studies that illustrate the main outcomes

Main outcomes	Quotes
Health related stigma by *employers* is a barrier for people with disabilities to sustainable employment and well-being at work	…In the end, it may not be certain what will happen when. They may not use their medication, may give up; who knows what they will do if conditions that trouble them start arising. Therefore, the company will not want to endanger its employees. (Employer) [32]
	Perhaps more than any other label in our society, having a serious mental illness indicates to the person and those around that s/he will never be capable of work. (Expert) [15]
	When I came back from being hospitalized, I was subtly discouraged from applying for a job that had been virtually promised to me prior to the hospitalization. (Person with mental illness) [37]
	I was having a lot of emotional difficulties and requested time off or a modified work schedule for medical reasons. My employer demanded a diagnosis so my therapist/psychiatrist provided the diagnosis. My employer denied the request and noted that if I had serious psychiatric issues then I needed to quit. (Person with mental illness) [37]
	I work in a grocery store. The manager is rough on most employees, but he puts additional pressure on me, even though I work as well as the others. One day he said: ‘don’t just sit there doing nothing, move a little for exercise. You would lose weight, and that would only do you good’. (Obese person) [77]
Health related stigma by *co-workers* is a barrier for people with disabilities to sustainable employment and well-being at work	With mental illness one has to work extra (like when one is any minority in this society) sometimes to prove one is capable of doing the same job as someone who isn’t a minority and mental illness is no exception. (Person with mental illness) [37]
	One girl made the comment she would not work for a crazy person. (Person with mental illness) [37]
	I have been socially and professionally marginalized, and expected to work without collegial support or interaction. (Person with mental illness) [37]
	‘‘I was able to handle all the gossip, but in the end I decided to choose for myself and go another way.’’ (HIV-positive person) [75]
Health related stigma by others: *health care professionals, reintegration professionals, customers, family and friends* is a barrier to sustainable employment	There’s an automatic assumption that someone’s disability is going to affect job performance, because people with disabilities have more glaring weaknesses – often their weaknesses are more apparent than other employees. (Employer referring to reintegration professionals/employers) [62]
	Because one barrier that I see in consumers that’s the most damaging is them being told you will never be able to work. And when you hear that from an authority that you recognize as an expert in mental illness, it has huge impact on how you see yourself. (Key informant talking about mental health professionals) [15]
	I find family members are a problem for our members. They do not want their kids to go back to work, for a couple of reasons. They are really concerned… (Service provider talking about people with mental illness) [91]
For *people with disabilities* anticipated stigma, self-stigma and ‘why try’ –effect, and the disclosure dilemma is a barrier to sustainable employment and well-being at work	I was aware that as soon as I ticked the psychiatric illness box I felt really that the door was closing… I think it was just that it was out of the question really, getting work, and saying to someone that I had a care program and medication. (Person with mental illness talking about the expectation that their application would be disregarded) [95]
	When you first find out you’ve got a mental illness … it’s like, you know, ‘you’re a retard’ type of thing. And then you’ve got the other extreme of all these people who are familiar with you for all these years, family, friends, etc., saying ‘there’s nothing wrong with you’. And so it’s like from one extreme to the other … you lose touch with yourself, with your own awareness of what you’re able to do … so that debilitates you.(Person with mental illness) [91]

### Employers

In several studies, employers were found to hold stigmatizing attitudes regarding employees with disabilities, which negatively affected disabled employees’ and job seekers’ well-being and sustainable employment. A main reason for this seemed to be a lack of knowledge of disabilities [13, 29–33]. This could be a lack of medical knowledge about the symptoms, re-occurrence, and recovery or a lack of knowledge about possible workplace accommodations [29, 34, 35]. One study showed that employers did not always understand that with reasonable workplace accommodation an employee with a disability could function the same as an employee without a disability [31]. Several studies mentioned the belief of employers that people with disabilities are incompetent to work; employers questioned the productivity, work abilities, and reliability of people with disabilities [30, 35–40]. In case of mental disabilities, studies found that employers had the presumption that emotional vulnerability affects cognitive capacity and decision-making ability and limits a worker’s ability to deal with stress or pressure [30, 37, 38]. Employers were found to be sensitive to concerns about the presumed incompetence of people with disabilities because they are responsible for achieving economic goals and business efficiency [15, 32]. Another belief was that people with disabilities could be dangerous [41]. Some employers feared that people with mental disabilities might be inclined to violence [32, 38]. Others feared that people with epilepsy are unpredictable, which can result in unsafe situations for the employee and others [42], and other employers feared that people with HIV/AIDS might be contagious and infect them or their employees [43, 44].

Health related stigma was often found to lead to discriminatory behaviours by employers, which made it challenging for people with disabilities to find or maintain sustainable employment. First, health related stigma negatively affected hiring decisions: a quarter of the selected studies showed that employers were reluctant to hire people with disabilities [17, 20, 30, 33, 37, 39, 42, 43, 47–61]. Once an employer was aware that a job candidate had a disability, the employer was inclined to focus more on the disability rather than on his/her actual abilities and skills [62, 63]. One study showed that people with mental disabilities who were looking for work reported higher levels of discrimination compared to working people with mental disabilities [33]. Second, health related stigma was found not only to be an obstacle in finding work, but also as a barrier in maintaining a job because of advancement-related discrimination, including limited promotions and training opportunities, denied raises, encouragement to retire or leave their jobs and wrongful dismissals [33, 35, 37, 50, 59, 64, 65]. Two studies showed that some employers also excluded these employees of certain work roles and responsibilities [34, 37], for example, a study where some officers gave soldiers with mental disabilities only a menial job, which these soldiers saw as an insult [37, 66]. People with disabilities sometimes experienced a lack of recognition and were forced to accept lower wages that did not reflect their performance [37]. Or they coped with negative reactions of employers to their disability by leaving their jobs [37, 67–71]. Other studies mentioned that leaving a job is not necessarily a voluntary act as some employers discharge employees due to (disclosing) their disability [36, 37, 47, 53, 54, 64, 72–75]. Third, stigma by employers was often found to be a barrier to maintaining a job either due to denying reasonable workplace accommodations or due to making it difficult for people with disabilities to access these accommodations [35, 37, 47, 59]. One study showed that people with invisible disabilities have more challenges accessing workplace accommodations compared to people with visible disabilities [60]. A lack of workplace accommodations made it hard for people with disabilities to meet the expectations of their employers [60]. Finally, stigma has been found to lead to negative consequences for the well-being at work of workers with disabilities due to bullying and harassment by employers [35, 47, 59]. A study showed that employers play an important role in creating a disability inclusive climate to decrease the perceived discrimination by disabled workers [76]. One study mentioned behaviours of employers due to stigma that, on the one hand, were mistrustful and cruel [19] and, on the other hand, were patronizing and overprotective [48, 65]. These behaviours made employees with disabilities feel upset and taken aback [70]. It also made them feel socially excluded by the employer, which for them led to a loss of motivation to perform the job [77].

### Co-workers

Several studies showed that co-workers, like employers, held stigmatizing attitudes towards people with disabilities, which resulted in adverse outcomes for the sustainable employment and well-being at work of workers with disabilities. This was due to a lack of knowledge and understanding of disabilities [13], which could lead to increased fearful and negative attitudes of co-workers at work. These attitudes negatively affected the ‘like-ability’ of adults with developmental disabilities [60]. Co-workers demonstrated a lack of belief that people with disabilities are competent to work [65, 78]. They had the same kind of concerns as employers had about the productivity, capacity to maintain work, reliability, and work abilities of people with disabilities [37, 79, 80]. In case of workers with cancer, co-workers believed the worker would perform poorly due to pain, lack of concentration, memory loss, post-treatment depression, and lack of confidence [81]. In case of mental disabilities, the supposed incompetence was due to the presumed emotional vulnerability that influenced a worker’s ability to handle stress [37]. Another negative belief was that workplace accommodations were perceived as unfair or as preferential treatment [78]. Workers are responsible for meeting a certain productivity standard together, any adjustments to workplace responsibilities can be seen as if the worker with a disability is not taking full responsibility for his/her part of the workload [15]. Co-workers thought that workers with a disability did not deserve the accommodation or should not be entitled to it; the emotional reaction of some co-workers to this was one of envy and resentment [37].

Health related stigma by co-workers can be a barrier for both well-being at work and sustainable employment. First, stigma by co-workers may lead to strained work relationships, which can make it hard for people with disabilities to maintain work. Stigma lowered the willingness of co-workers to work with people with disabilities [78]. Co-workers tended to focus on the disability rather than the work abilities and skills [63]. The belief that people with disabilities are incompetent played an important role in why they were having a less favourable attitude towards working with a people with a disability [37, 80]. Co-workers sometimes didn’t want to collaborate with people with disabilities and do work for them, or they might exclude them within the context of work roles and responsibilities [37]. In addition, co-workers held them accountable for their work to a different extent due to the disability. Some co-workers made them feel that they had to work harder to show that they could manage their jobs the same as a person without a disability, ignoring their limitations [37]. Or co-workers were seen as over-protective, insinuating that people with disabilities are not capable of performing the job [37, 65]. The unequal treatment by co-workers could lead to feelings of frustration, increased tension, and strained relationships between co-workers and workers with disabilities, which made it hard for people with disabilities to maintain a job [60]. Furthermore, health related stigma can lead to harassment and bullying, which negatively influences the well-being at work and can prompt the disabled worker to resign. One study about people with HIV identified gossip as negative behaviour of co-workers [75]. Another study, focused on mental disabilities, mentioned the use of insensitive, derogatory or disrespectful language and of the mental disabilities being used as leverage causing workers to change the way they act [37]. Two studies showed that health related stigma led mistrustful and cruel behaviour by co-workers[65, 66]. These negative responses of co-workers had a negative impact on the well-being of workers with disabilities [82, 83]. It could make them feel stressed or unsafe [75], upset and taken aback [70], and it could lower their self-esteem through internalized stigma [84]. Negative responses could prompt people with disabilities to resign [75]. Finally, health related stigma by co-workers could result in social exclusion, which led to a diminished well-being at work of people with disabilities and problems in maintaining a job. Studies about both physical and mental disabilities reported social exclusion by co-workers [37, 48, 60, 66, 77, 85–88]. However, the risk of social exclusion seemed to be especially prominent in case of workers with contagious diseases like HPB/C or HIV due to the concerns of co-workers about possible transmission. Co-workers with concerns about transmission were less likely to accept their colleagues [89, 90]. The less willing a co-worker was to accept a worker the more they tend to avoid contact with this person, even after a risk assessment took place [87]. Co-workers could even request a job change when a worker was diagnosed as HIV-positive [88]. Strained relationships caused by social exclusion [60] could lead to a loss of motivation to perform the job [77].

### Stakeholders Outside the Work Context

Stakeholders outside of the direct workplace context also play a role in how health related stigma affects sustainable employment of people with disabilities. For instance, some mental health professionals were found to play a discouraging role. They tended to focus on the medical perspective, which means that someone’s deficits get more attention than someone’s work abilities [15]. Some mental health professionals thought that people with mental illness are incapable of work [15], or they tended to value therapy or medications over work, which results in limited work expectations [91] and a lack of encouragement to work [15, 92]. Although health care professionals mainly played a role in studies focusing on mental health problems, one study showed that health professionals could play a similar discouraging role when it comes to a physical disability like Multiple Sclerosis [93]. People with disabilities tended to internalize what health care professionals said in regard to their work ability [94], which could result in not pursuing a job or quitting a job. A second stakeholder group whose behaviour could act as a barrier to sustainable employment of disabled workers were reintegration professionals. Their stigmatizing attitudes decreased the chance of being referred to a position because reintegration professionals thought a client was unable to handle the position [62]. A third stakeholder group were customers, who can affect the sustainable employment of people with disabilities in a different way. One study on obesity showed that stigma by customers could be harmful for maintaining work because it lead to negative evaluations of the employee [56]. Finally, attitudes of family and friends could also be a barrier to employment [91]. Family could offer emotional support and help in making career decisions, but they sometimes feared the stress of work and giving up benefits and, therefore, did not encourage their disabled loved ones to get a job.

### People with Disabilities

Findings of several studies showed that people with disabilities can be reluctant in finding and maintaining sustainable employment and their well-being at work. In the first place, anticipated stigma was found to discourage people with disabilities to pursue employment or maintain employment. Studies showed that many people with disabilities accepted being discriminated [92, 95]. They feared that their application would be discarded [83, 95], that they would be fired [48, 83], that at least some co-worker would discriminate them, that they would lose health care benefits [53, 83], or that they would have limited promotion opportunities [83]. Past stigma experiences related to the fear of workplace discrimination [96, 97]. Anticipated stigma stopped people with disabilities from applying for work [53, 61, 73, 77, 98–103] or applying for education or training [61, 101, 102]. In maintaining work, anticipated stigma could make people avoid the chance of promotion [104], or it could lead to non-disclosure [33], which lowered the chance of getting necessary workplace support [19, 31]. Moreover, self-stigma and the ‘Why Try’ effect could lead to insufficient motivation and effort to find or maintain employment and decreases well-being at work. Self-stigma is a process in which people with disabilities internalize negative stereotypes, which resulted in losing sight of their work abilities and potential [62, 69, 91, 92, 105] and a lower self-esteem [84, 105]. One study showed the vicious circle where a job was necessary for improving the self-esteem of people with disabilities, but a job had not been within reach due to their low self-esteem [106]. Self-stigma undermined the motivation of people with disabilities to aspire, secure, or maintain employment [15, 92, 96] and negatively affected their self-efficacy [107]. If people with disabilities were not motivated to undertake action to maintain or find employment due to self-stigma, it was challenging to find or maintain a job [24, 39, 108, 109], e.g., due to avoidance of the prospect of promotion [104], less career achievement or advancement [69], earning less [110], not returning to work [106], dropping out, or changing career-goals [107]. Self-stigma did not only affect sustainable employment but it could also affect well-being at work because self-stigma could lead to lower help-seeking intentions [15, 66, 111–113] although one study did not confirm this [114]. Lower help-seeking intentions were associated with more stress and burnout [22, 115]. Furthermore, whether to disclose a disability or not at the workplace is a stigma-related, difficult and personal decision that might have consequences for someone’s sustainable employment and well-being at work. Disclosure could make it easier to perform the job by getting access of accommodations [19, 20, 82, 116] and through better understanding, compassion, and practical support of colleagues [64, 72]. Disclosure could also reduce or diminish the stress associated with concealment; it made people able to be their authentic selves, which was important for their overall well-being [64]. In contrast, disclosure could lead to adverse outcomes like stigma and discrimination [116]. While the stress related to the concealment was reduced, the overall stress level increased and the overall well-being of people with disabilities was less positive because of the experience of these negative outcomes [82]. On the other hand, non-disclosure allowed a person with a disability to perform the job without fear of stigma and discrimination [19]. Non-disclosure was, however, not only associated with positive employment outcomes like predicted reemployment [117], it could also lead to job loss [60].

## Discussion

### Summary of the Findings

The aim of this systematic review was to analyse studies that have addressed ways in which stigma affects sustainable employment and well-being at work of people with disabilities. Many of the 96 studies included in this review focussed on the role of employers. For example, employers often displayed formal discrimination, such as hiring discrimination or advancement-related discrimination. There was also evidence for bullying, harassment, and social discrimination by employers. However, these behaviours were more likely to be displayed by co-workers and should not be underestimated in their adverse outcomes for people with disabilities in maintaining work and well-being at work. In addition, studies showed that employment outcomes could be influenced by anticipated stigma, self-stigma and the ‘why try’ effect, and the disclosure dilemma as a result of negative attitudes and behaviours of others. This review also found evidence for the role of other stakeholders, especially health care professionals, reintegration professionals, co-workers, and family and friends in discouraging people with disabilities to pursuit or maintain employment. However, this review also shows that the effects of these stakeholders’ attitudes and behaviours on well-being and sustainable employment of people with health problems in particular are understudied and urgently need more attention.

This systematic review shows that there are many similarities in the ways in which health related stigma affects sustainable employment and well-being at work, regardless of the type of the mental or physical disability. However, several nuances were found. For example, health related stigma was more prominent concerning infectious diseases such as HIV/AIDS due to employers’ and co-workers' fears of being infected compared to other physical or psychological diseases. In case of mental disabilities, employers and co-workers were sensitive to concerns about the emotional vulnerability that might affect someone’s cognitive capacity, decision-making ability, and ability to deal with stress or pressure.

### Strengths and Limitations

The strength of this systematic review is that the ways in which stigma affects sustainable employment and well-being at work were researched, regardless of the type of the mental or physical disability. The inclusion of quantitative as well as qualitative studies and mixed method studies strengthens the findings of this review. Furthermore, searches from six different databases were conducted. All articles were screened and assessed by at least two people simultaneously and independently.

Although the literature was systematically searched, it is possible that relevant studies were not found or included. Articles for which no abstract and no full text was available were excluded from the search only after multiple unsuccessful attempts to find the item (e.g., searching multiple databases and two attempts to contact the author). Another limitation was the restriction of the search results to articles in English, as it is possible that potential cultural contexts were missed.

## Conclusion

The literature seems to increasingly pay attention to health related stigma within the work context since almost half of the selected studies were published between 2015 and 2019. However, many of the findings from the selected studies did not arise from studies that directly addressed health related stigma in relation to sustainable employment and well-being at work. Therefore, future empirical research should focus directly on health related stigma and how it affects occupational outcomes. Additionally, since we have found several nuances in which health related stigma affects sustainable employment and well-being at work regarding the type of the mental or physical disability, more in-depth research on how these themes interlink for each disability area is needed.

Most studies on health related stigma have been conducted in Western countries. Evidence shows that, in Western countries, the ways in which stigma affects sustainable employment and well-being at work have commonalities but can be slightly different in non-Western countries [118, 119]. It is, therefore, important to investigate how health related stigma influences occupational outcomes in non-Western countries.

Previous review articles focused on the role of employers, co-workers, and people with disabilities themselves regarding stigma and work [120, 121]. Since we have found that stakeholders outside the direct work context, such as health care professionals [15, 91–94], reintegration professionals [62], customers [56], family and friends [91], also play an important role, more research is needed on the stigmatizing attitudes and behaviours of these stakeholders and on how it affects sustainable employment and well-being at work. The findings of this review can contribute to extending theoretical knowledge in order to develop validated measures of health related stigma by different stakeholders, contextualized in the work context, as these are scarce [122] but much needed to evaluate the consequences of health related stigma for sustainable employment and well-being at work [8].

In addition, the knowledge obtained in this review contributes to the development of interventions focusing on improving the position on the labour market for people with mental or physical health problems. Destigmatizing intervention studies have been conducted. Many of these studies pay attention to mental health stigma [120].There are a few intervention studies concerning physical health stigma regarding HIV [123] and epilepsy [124].

Many destigmatizing interventions focus on the role of employers, co-workers, or people with a disability themselves [120]. To our knowledge, hardly any interventions focus on health related stigma in the workplace by stakeholders outside the direct work context. There are some interventions that could be used for that purpose although reducing stigma in the work context is not the primary aim of these interventions [120].

Future interventions should focus directly on both stakeholders inside and outside the workplace context in order to promote sustainable employment and well-being at work for people with both mental and physical health problems. This will not only lead to positive consequences regarding the (mental) health of individuals but also to positive economic and societal outcomes [8, 125].

## Supplementary Information

Below is the link to the electronic supplementary material.

Supplementary file1 (DOCX 15 KB)

Supplementary file2 (DOCX 13 KB)

Supplementary file3 (DOCX 40 KB)

## Data Availability

The research material is available on request.
