# Supplementary material for: In What Ways Does Health Related Stigma Affect Sustainable Employment and Well-Being at Work? A Systematic Review
Source: J Occup Rehabil. 2021 Sep 6;32(3):365–79. doi: 10.1007/s10926-021-09998-z (PMC9576674; doi:10.1007/s10926-021-09998-z)
Supplement: Supplementary file 1 — Supplementary file1 (DOCX 15 KB) [file 10926_2021_9998_MOESM1_ESM.docx]

**Online Appendix 1. Search strategy**

**Embase**

('stigma'/de OR 'social stigma'/de OR 'stereotyping'/de OR stereotypy/de OR 'social distance'/de OR 'social discrimination'/de OR 'social exclusion'/de OR 'employment discrimination'/de OR 'self disclosure'/de OR (stigma* OR stereotyp* OR (social* NEAR/3 (distan* OR percept* OR exclusion*)) OR discrimination* OR discriminated* OR shame):ab,ti OR (disclosure* OR nondisclosure*):ti) AND ('diseases'/de OR 'mental disease'/de OR 'mental disease'/exp/mj OR 'physical disease'/de OR 'physical disease'/exp/mj OR 'chronic disease'/de OR 'disabled person'/de OR 'mental patient'/de OR 'mental health'/de OR patient/exp/mj OR 'burnout'/de OR 'attitude to disability'/de OR (health-related OR unhealthy* OR disabilit* OR disabeled* OR injur* OR patient* OR disease* OR illness* OR mental-health OR burnout OR burn*-out* OR disorder* OR ill):ab,ti) AND ('employment discrimination'/de OR 'employment'/exp OR employee/de OR employer/de OR 'job performance'/de OR 'occupational health'/de OR employability/de OR 'workplace'/de OR 'job finding'/de OR 'job interview'/de OR 'employee attitude'/de OR 'vocational rehabilitation'/de OR 'absenteeism'/de OR 'presenteeism'/de OR 'medical leave'/de OR 'return to work'/de OR 'career'/de OR 'job stress'/exp OR (employment* OR unemploy* OR ((work OR worker* OR workforce OR job OR career* OR occupation*) NEAR/3 (perform* OR interview* OR search OR seeking OR loss OR finding OR well-being OR wellbeing OR stress OR affect* OR discomfort* OR effectiv* OR barrier* OR facilitator*)) OR (professional* NEAR/3 (effectiv* OR well-being OR wellbeing)) OR employab* OR employer* OR employee* OR ((occupation* OR vocational*) NEAR/3 (health* OR impact* OR rehabilit*)) OR (work NEAR/3 (resum* OR return*)) OR workplace* OR ((labour OR labor) NEAR/3 market*) OR hiring OR firing OR absenteeism* OR presenteeism* OR ((sick OR medical) NEAR/3 leave*)):ab,ti) NOT ([Conference Abstract]/lim) AND [English]/lim NOT ([animals]/lim NOT [humans]/lim) NOT (juvenile/exp NOT adult/exp)

**Medline Ovid**

(Social Stigma/ OR Stereotyping/ OR Social Distance/ OR Social Discrimination/ OR Social Perception/ OR Self Disclosure/ OR Disclosure/ OR (stigma* OR stereotyp* OR (social* ADJ3 (distan* OR percept* OR exclusion*)) OR discrimination* OR discriminated* OR shame).ab,ti. OR (disclosure* OR nondisclosure*).ti.) AND (*Disease/ OR Mental Disorders/ OR exp *Mental Disorders/ OR exp * Diseases Category/ OR Chronic Disease/ OR Disabled Persons/ OR Mentally Ill Persons/ OR Mental Health/ OR exp *Patients/ OR Burnout, Psychological/ OR exp *"Bacterial Infections and Mycoses"/ OR exp *Cardiovascular Diseases/ OR exp *Chemically-Induced Disorders/ OR exp *"Congenital, Hereditary, and Neonatal Diseases and Abnormalities"/ OR exp *Digestive System Diseases/ OR exp *Disorders of Environmental Origin/ OR exp *Endocrine System Diseases/ OR exp *Eye Diseases/ OR exp *"Female Urogenital Diseases and Pregnancy Complications"/ OR exp *"Hemic and Lymphatic Diseases"/ OR exp *Immune System Diseases/ OR exp *Male Urogenital Diseases/ OR exp *Musculoskeletal Diseases/ OR exp *Neoplasms/ OR exp *Nervous System Diseases/ OR exp *"Nutritional and Metabolic Diseases"/ OR exp *Occupational Diseases/ OR exp *Otorhinolaryngologic Diseases/ OR exp *Parasitic Diseases/ OR exp *"Pathological Conditions, Signs and Symptoms"/ OR exp *Respiratory Tract Diseases/ OR exp *"Skin and Connective Tissue Diseases"/ OR exp *Stomatognathic Diseases/ OR exp *Virus Diseases/ OR exp *"Wounds and Injuries"/ OR (health-related OR unhealthy* OR disabilit* OR disabeled* OR injur* OR patient* OR disease* OR illness* OR mental-health OR burnout OR burn*-out* OR disorder* OR ill).ab,ti.) AND (Employment/ OR Work Performance/ OR Occupational Health/ OR Workplace/ OR Rehabilitation, Vocational/ OR Absenteeism/ OR Presenteeism/ OR Return to Work/ OR Occupational Stress/ OR (employment* OR unemploy* OR ((work OR worker* OR workforce OR job OR career* OR occupation*) ADJ3 (perform* OR interview* OR search OR seeking OR loss OR finding OR well-being OR wellbeing OR stress OR affect* OR discomfort* OR effectiv* OR barrier* OR facilitator*)) OR (professional* ADJ3 (effectiv* OR well-being OR wellbeing)) OR employab* OR employer* OR employee* OR ((occupation* OR vocational*) ADJ3 (health* OR impact* OR rehabilit*)) OR (work ADJ3 (resum* OR return*)) OR workplace* OR ((labour OR labor) ADJ3 market*) OR hiring OR firing OR absenteeism* OR presenteeism* OR ((sick OR medical) ADJ3 leave*)).ab,ti.) NOT (news OR congres* OR abstract* OR book* OR chapter* OR dissertation abstract*).pt. AND english.la. NOT (exp animals/ NOT humans/) NOT ((exp child/ OR exp infant/ OR pediatrics/ OR adolescent/) NOT exp adult/)

**PsycINFO Ovid**

(Stigma/ OR Stereotyped Attitudes / OR Social Discrimination/ OR Social Perception/ OR Self-Disclosure/ OR (stigma* OR stereotyp* OR (social* ADJ3 (distan* OR percept* OR exclusion*)) OR discrimination* OR discriminated* OR shame).ab,ti. OR (disclosure* OR nondisclosure*).ti.) AND (Mental Disorders/ OR exp *Mental Disorders/ OR Chronic Illness / OR "Disabled (Attitudes Toward)" / OR "Mental Illness (Attitudes Toward)" / OR Mental Health/ OR exp *Patients/ OR exp * Cardiovascular Disorders / OR exp * " Congenital Disorders "/ OR exp * Eye Disorders/ OR exp *" Urinary Function Disorders "/ OR exp * Urogenital Disorders / OR exp * Musculoskeletal Disorders / OR exp *Neoplasms/ OR exp * Nervous System Disorders / OR exp *"Metabolism Disorders"/ OR exp * Parasitic Disorders / OR exp * Respiratory Tract Disorders / OR exp * Viral Disorders / OR exp *"Wounds"/ OR (health-related OR unhealthy* OR disabilit* OR disabeled* OR injur* OR patient* OR disease* OR illness* OR mental-health OR burnout OR burn*-out* OR disorder* OR ill).ab,ti.) AND (exp Employment Status / OR Job Performance / OR Occupational Health/ OR Diversity in the Workplace / OR Vocational Rehabilitation / OR Employee Absenteeism / OR Employee Productivity / OR Reemployment / OR Occupational Stress/ OR (employment* OR unemploy* OR ((work OR worker* OR workforce OR job OR career* OR occupation*) ADJ3 (perform* OR interview* OR search OR seeking OR loss OR finding OR well-being OR wellbeing OR stress OR affect* OR discomfort* OR effectiv* OR barrier* OR facilitator*)) OR (professional* ADJ3 (effectiv* OR well-being OR wellbeing)) OR employab* OR employer* OR employee* OR ((occupation* OR vocational*) ADJ3 (health* OR impact* OR rehabilit*)) OR (work ADJ3 (resum* OR return*)) OR workplace* OR ((labour OR labor) ADJ3 market*) OR hiring OR firing OR absenteeism* OR presenteeism* OR ((sick OR medical) ADJ3 leave*)).ab,ti.) NOT (news OR congres* OR abstract* OR book* OR chapter* OR dissertation abstract*).pt. AND english.la. NOT (exp animals/ NOT humans/) NOT ((100.ag) NOT 300.ag.)

**Web of science Core Collection**

((TS=(stigma* OR stereotyp* OR (social* NEAR/2 (distan* OR percept* OR exclusion*)) OR discrimination* OR discriminated* OR shame) OR TI=(disclosure* OR nondisclosure*)) AND (TS=(health-related OR unhealthy* OR disabilit* OR disabeled* OR injur* OR patient* OR disease* OR illness* OR mental-health OR burnout OR burn*-out* OR disorder* OR ill)) AND (TS=(employment* OR unemploy* OR ((work OR worker* OR workforce OR job OR career* OR occupation*) NEAR/2 (perform* OR interview* OR search OR seeking OR loss OR finding OR well-being OR wellbeing OR stress OR affect* OR discomfort* OR effectiv* OR barrier* OR facilitator*)) OR (professional* NEAR/2 (effectiv* OR well-being OR wellbeing)) OR employab* OR employer* OR employee* OR ((occupation* OR vocational*) NEAR/2 (health* OR impact* OR rehabilit*)) OR (work NEAR/2 (resum* OR return*)) OR workplace* OR ((labour OR labor) NEAR/2 market*) OR hiring OR firing OR absenteeism* OR presenteeism* OR ((sick OR medical) NEAR/2 leave*)))) AND DT=(article) AND LA=(english)

**Cochrane CENTRAL**

((stigma* OR stereotyp* OR (social* NEAR/3 (distan* OR percept* OR exclusion*)) OR discrimination* OR discriminated* OR shame):ab,ti OR (disclosure* OR nondisclosure*):ti) AND ((health next related OR unhealthy* OR disabilit* OR disabeled* OR injur* OR patient* OR disease* OR illness* OR mental next health OR burnout OR burn* next out* OR disorder* OR ill):ab,ti) AND ((employment* OR unemploy* OR ((work OR worker* OR workforce OR job OR career* OR occupation*) NEAR/3 (perform* OR interview* OR search OR seeking OR loss OR finding OR well next being OR wellbeing OR stress OR affect* OR discomfort* OR effectiv* OR barrier* OR facilitator*)) OR (professional* NEAR/3 (effectiv* OR well next being OR wellbeing)) OR employab* OR employer* OR employee* OR ((occupation* OR vocational*) NEAR/3 (health* OR impact* OR rehabilit*)) OR (work NEAR/3 (resum* OR return*)) OR workplace* OR ((labour OR labor) NEAR/3 market*) OR hiring OR firing OR absenteeism* OR presenteeism* OR ((sick OR medical) NEAR/3 leave*)):ab,ti)

**Google scholar**

stigma|stereotypy|"social distance|exclusion|discrimination" "health-related"|unhealthy|disabilities|disabeled|injuries|patient|diseases|illness|"mental-health" employment| employability|unemployed|"work|job performance|wellbeing|stress"|employer|employee
