# Supplementary material for: In What Ways Does Health Related Stigma Affect Sustainable Employment and Well-Being at Work? A Systematic Review
Source: J Occup Rehabil. 2021 Sep 6;32(3):365–79. doi: 10.1007/s10926-021-09998-z (PMC9576674; doi:10.1007/s10926-021-09998-z)
Supplement: Supplementary file 2 — Supplementary file2 (DOCX 13 KB) [file 10926_2021_9998_MOESM2_ESM.docx]

**Online Appendix 2. Methodological quality criteria**

1. Qualitative
   1. Qualitative objective or question
   2. Appropriate qualitative approach or design or method
   3. Description of the context
   4. Description of participants and justification of sampling
   5. Description of qualitative data collection and analysis
   6. Discussion of researchers’ reflexivity
2. Quantitative experimental
   1. Appropriate sequence generation and/or randomization
   2. Allocation concealment and/or blinding
   3. Complete outcome data and/or low withdrawal/drop-out
3. Quantitative observational
   1. Appropriate sampling and sample
   2. Justification of measurements (validity and standards)
   3. Control of confounding variables
4. Mixed methods
   1. Justification of the mixed methods design
   2. Combination of qualitative and quantitative data collection-analysis techniques or procedures
   3. Combination of qualitative and quantitative data collection-analysis techniques or procedures
