# Supplementary material for: In What Ways Does Health Related Stigma Affect Sustainable Employment and Well-Being at Work? A Systematic Review
Source: J Occup Rehabil. 2021 Sep 6;32(3):365–79. doi: 10.1007/s10926-021-09998-z (PMC9576674; doi:10.1007/s10926-021-09998-z)
Supplement: Supplementary file 3 — Supplementary file3 (DOCX 40 KB) [file 10926_2021_9998_MOESM3_ESM.docx]

**Online Appendix 3. Table 1**

**Table 1** Characteristics of included studies (N=96)

| Country | Author (year) | Target health problem | Study population | Objective | Design |
| --- | --- | --- | --- | --- | --- |
| USA | Ameri et al. (2018)^[17]^ | Mental and physical: SCI and Asperger’s syndrome | N=6016 (fake) applications sent out for job applications, employers evaluated these applications | To investigate potential discrimination against people with disabilities. | Quantitative study, fake job applications send out to real job opportunities. |
| USA | Bautista & Wludyka (2006)^[98]^ | Physical: epilepsy | N=262 patients in epilepsy center | To determine those variables associated with employment in patients followed at a level 4 epilepsy center. | Quantitative study using surveys. |
| USA | Britt et al. (2019)^[111]^ | Mental | N=349 (time 1) N=112 (time 2) active-duty military personnel | To examine the perceived unit climate of support for mental health as a predictor of perceived stigma. | Quantitative longitudinal study using surveys |
| USA | Brown & Bruce (2016)^[112]^ | Mental | N=276 soldiers and veterans of Operation Enduring Freedom and Operation Iraqi Freedom | To increase our understanding of low treatment-seeking rates among soldiers and veterans by expanding upon previous measurements of the stigma construct and examining factors influencing willingness to seek treatment. | Quantitative study using online surveys. |
| USA | Chan et al. (2005)^[47]^ | Mental and physical | N=35.763 allegations of discriminations filed by people with disabilities | To determine what drives workplace discrimination against people with disabilities. | Quantitative study with a descriptive design, an ex post facto design to examine allegations of discrimination. |
| USA | Chang (2015)^[86]^ | Mental: SMI | N=2 persons with SMI | To demonstrate the challenges that two individuals with SMI had experienced in competitive employment settings. | Qualitative study using interviews. |
| USA | Crom et al. (2017)^[67]^ | Physical: cancer | N=289 adult survivors of childhood cancer with a history of employment | To investigate workplace experiences and turnover intention and to examine factors associated with turnover intention among survivors. | Quantitative study where participants completed measures of workplace experiences. |
| USA | Dalgin & Bellini (2008)^[57]^ | Mental and physical: insulin-dependent diabetes and bipolar disorder | N=60 employers | To investigate the impact of disclosure of invisible disabilities (physical and psychiatric) within the employment interview process. | Quantitative study, experimental study using vignettes to let employers make hiring decisions where the disability type and extent of disclosure were manipulated. |
| USA | Dalgin & Gilbrede (2003)^[48]^ | Mental | N=11 people who self-identified as having psychiatric disabilities/labels | To gather data from people with psychiatric disabilities/labels regarding employment disclosure. | Qualitative study including a focus group and individual interviews. |
| USA | Dolce et al. (2018)^[30]^ | Mental | N=14 human resource officers | To examine human resource professionals’ views on hiring and employing individuals with psychiatric disabilities. | Qualitative study using focus groups. |
| USA | Frndak et al. (2015)^[116]^ | Physical: MS | N=246 employed patients with MS | To determine clinical factors most predictive of disclosure, and to measure the effects of disclosure on workplace problems and accommodations in employed patients. | Mixed methods study including a comparison of a cross-section sample, a longitudinal sample and a case study. |
| USA | Gerlach et al. (2017)^[110]^ | Physical: stuttering | N=261 people who stutter | To quantify relationships between stuttering and labour market outcomes, determine if outcomes differ by gender, and explain the earnings difference between people who stutter and people who do not stutter. | Mixed method study using surveys and interviews. |
| USA | Goldberg et al. (2005)^[19]^ | Mental | N=32 people who have experienced significant symptoms of psychiatric disabilities | To explore the complex situations around employment and disclosure. | Qualitative study using interviews. |
| USA | Henry & Lucca (2003)^[91]^ | Mental: SMI | N=44 adults with SMI, N=30 employment service providers | To examine the perspectives of people with psychiatric disabilities and employment service providers regarding factors that most directly help or hinder consumer efforts to obtain and maintain employment. | Qualitative study using focus groups. |
| USA | Henry et al. (2014)^[62]^ | Mental and physical | N=74 private and public sector employers | To examine employers’ perspective related to challenges they face when hiring people with disabilities. | Qualitative study using focus groups. |
| USA | Hernandez et al. (2008)^[50]^ | Mental and physical | N=21 employers | The purpose of this qualitative study was to explore the experiences of employers with workers with disabilities. | Qualitative study using focus groups. |
| USA | Hipes et al. (2016)^[51]^ | Mental | N=635 resumes and cover letters of a fake applicant with a history with mental illness were sent to 635 jobs | To extend research on reactions to mental illness by utilizing a field experiment to test effects of mental illness labels on labour market discrimination. | Qualitative study using an experimental design. |
| USA | Isetti et al. (2017)^[52]^ | Physical: ADSD | N=32 human resource personnel members | To determine the influence of symptom severity and disclosure of ADSD on the perceptions of human resource personnel members during a simulated phone job interview. | Qualitative study using an experimental design. |
| USA | McBee-Black & Ha-Brookshire (2018)^[107]^ | Mental and physical | N=12 people living with disabilities | To explore how the barriers to social participation, specifically workplace participation, faced by people living with disabilities, are exacerbated by the lack of appropriate clothing and the role that stigma, self-efficacy, and clothing have in workplace participation. | Qualitative study, using interviews. |
| USA | Mclaughlin et al. (2004)^[78]^ | Physical: AIDS, CP and stroke | N=657 undergraduate students enrolled in organizational behaviour courses | To evaluate the roles of disability type (AIDS, CP and stroke), stigma, and employee characteristics in acceptance of a co-worker with a disability. | Qualitative study using an experimental design. |
| USA | Mullen & Crowe (2016)^[22]^ | Mental: burnout and stress | N=333 school counsellors | To investigate the relationship between self-stigma, help seeking, satisfaction with life, burnout, and stress with a sample of practicing school counsellors. | Quantitative study using surveys (path analysis). |
| USA | Perry et al. (2000)^[55]^ | Physical | N=301 college graduates with disabilities, N=329 college graduates without disabilities. | To explore the extent to which college graduates with and without physical disabilities reported experiencing discrimination in their overall work histories. | Qualitative study using questionnaires that were analysed using qualitative methods. |
| USA | Randolph (2005)^[65]^ | Mental and physical | N=3 women with various disabilities | To describe how women with disabilities who are in the workplace experience discrimination. | Qualitative study using interviews. |
| USA | Rao et al. (2010)^[41]^ | Mental and physical: HIV, psychosis, bone cancer, drug dependence and alcohol dependence | N=879 employers from Beijing, Hong Kong and Chicago. | To examine whether the construct of individualism can help to explain cross-cultural differences in stigmatizing attitudes observed between American and Chinese employers. | Quantitative study using surveys. |
| USA | Reed et al. (2017)^[74]^ | Physical: MS | N=72 workers with MS | To identify factors that may influence the decision to disclose disability to an employer and the consequences of disclosure for participants with MS. | Qualitative study using focus groups. |
| USA | Ruggs et al. (2015)^[56]^ | Physical: obesity | N=6 confederates acting as job applicants, customers, observers and store personnel | To examine issues related to the influence of weight-based stigmatization in retail settings. | Quantitative study using questionnaires. |
| USA | Russinova et al. (2011)^[37]^ | Mental: SMI | N=436 individuals with SMI | To present ﬁndings about the manifestations of prejudice and discrimination at the workplace. | Quantitative study using surveys. |
| USA | Scheid (2005)^[38]^ | Mental | N=190 employers | To examine the role played by stigma in employers’ response to the 1990 Americans with Disability Act (ADA) | Quantitative study, using surveys and telephone interviews. |
| USA | Spiegel et al. (2016)^[82]^ | Physical: degenerative eye condition | N=143 individuals with degenerative eye conditions | To investigate when the disclosure of a degenerative eye condition relates positively to well-being. | Mixed methods design, using survey data (pathway analysis) and using qualitative life stories interview (pathway analysis). |
| USA | von Schrader et al. (2013)^[83]^ | Mental and physical | N=780 individuals with disabilities | To present the findings of a survey of individuals with disabilities focused on identifying and better understanding the factors that influence the disclosure decision. | Quantitative study using surveys. |
| CAN | Benoit et al. (2013)^[108]^ | Physical: legally blind | N=41.750 legally blind people, N= 18 legally blind people | This study aims to explore whether people with disabilities are able to practice their rights against discrimination. | Mixed methods design, quantitative survey and in-person interviews. |
| CAN | Boychuk et al. (2018)^[109]^ | Mental: psychosis | N=36 young adults experiencing first-episode psychosis and currently receiving treatment from an early intervention team and having work or education as a goal. | To advance a model of the career decision-making processes of young adults with first-episode psychosis, and the influences that affect their career decision-making. | Qualitative study, a grounded theory study. Involving a demographic questionnaire, which was followed-up by a one-on-one interview based on a semi structured interview protocol. |
| CAN | Hand & Tryssenaar (2006)^[49]^ | Mental | N=58 small business employers | To investigate the beliefs of small business employers regarding hiring individuals with mental illness. | Mixed methods study using surveys, followed by in-person interviews and self-administered questionnaires. |
| CAN | Krupa et al. (2009)^[15]^ | Mental | N=500 Canadian documents N=19 key informants | To advance the development of theory related to the stigma of mental illness in employment to serve as a guiding framework for intervention approaches. | Quantitative study using a constructivist grounded theory methodology to analyse documents and interviews. |
| CAN | Lindsay et al. (2019)^[31]^ | Mental and physical | N=17 youth with disabilities aged 15-34 | To explore when and how young people with disabilities disclose their condition and request workplace accommodations. | Qualitative study using interviews. |
| CAN | Lindsay et al. (2018)^[63]^ | Physical | N=35 18 employers who hire people with disabilities; 17 employees with a disability | To explore the concept of disability confidence from two perspectives, employers who hire people with a disability and employees with a disability. | Qualitative study using interviews. |
| CAN | Mansfield et al. (2015)^[34]^ | Physical: wrMTBI | N=12 workers with wrMTBI | To explore how individuals with wrMTBI experience return-to-work processes when returning to the workplace where the injury occurred. | Qualitative study using interviews. |
| CAN | Michalak et al. (2007)^[64]^ | Mental: Bipolar disorder | N=52 people with bipolar disorder | To provide a detailed description of the different ways in which bipolar disorder can impact upon occupational functioning. | Qualitative study using interviews. |
| CAN | Oud (2019)^[79]^ | Mental and physical | N=10 academic librarians with disabilities | To broaden understanding of an overlooked minority group in the profession and to discover what, if any, equity issues librarians with disabilities face at work. | Qualitative study using interviews. |
| CAN | Poremski et al. (2015)^[73]^ | Mental | N=27 recently housed adults with mental illness | To explore which barriers to employment, directly resulting from having been homeless persisted once stable housing was provided to individuals with a diagnosis of mental illness. | Qualitative study using interviews. |
| CAN | Stergiou-Kita et al. (2017)^[59]^ | Physical: severe brain injury | N=10 persons with brain injuries, N=5 employment service providers | To explore how stigma may inﬂuence return-to-work processes, experiences of stigma and discrimination at the workplace for persons with brain injuries, and strategies that can be employed to manage disclosure. | Qualitative study using interviews. |
| CAN | Stergiou-Kita et al. (2016)^[35]^ | Physical: cancer | N=16 cancer survivors, N=16 health care/vocational service providers, N=8 employer representatives | To examine how stigma develops in work contexts and how it influences cancer survivors' return to work process and their disclosure decisions. | Qualitative study using interviews. |
| CAN | Teindl et al. (2018)^[60]^ | Physical: developmental disabilities | N=24 employment support professionals, N=8 caregivers and N=14 persons with developmental disabilities | To explore how visibility of a disability inﬂuences employment for adults with developmental disabilities. | Qualitative study using interviews. |
| CAN | Toth & Dewa (2014)^[113]^ | Mental | N=13 employees of a post-secondary educational institution | To develop a model of disclosure specific to mental health issues in a work context. | Qualitative study using interviews. |
| AUS | Allen & Carlson (2003)^[45]^ | Mental and physical | N=13 adults with a disability arising from an illness or injury with consequent losses in their work capacity | To explore the theme of concealment in the disability-to-employment transition in detail. | Qualitative study using interviews. |
| AUS | Bricker-Katz et al. (2013)^[104]^ | Physical: stuttering | N=9 people who stutter | To examine the experimental claims of nine people who stuttered with the purpose of determining the impact of stuttering on their work lives and to further examine what meaning they derive from these experiences. | Qualitative study using interviews. |
| AUS | Gladman & Waghorn (2016)^[36]^ | Mental: SMI | N= 39 persons with psychotic disorder | To increase understanding of personal experiences of employment and how these experiences can be used to inform the assistance provided in support of client' competitive employment goals. | Qualitative study using an experimental design. |
| AUS | Hielscher & Waghorn (2017)^[96]^ | Mental: SPMI | N=25 patients diagnosed with a SPMI and current volunteer for vocational rehabilitation assistance or having a vocational goal. | To develop and trial brief measures of employment values and employment fears that could be used to further investigate any impacts of community stigma on personal employment goals. | Quantitative study, exploring psychometric properties of a new Employment Fears Scale and an Employment Values Scale. |
| AUS | Kirk-Brown & van Dijk (2014)^[69]^ | Physical: MS | N=40 employees with MS | To identify the types of psychosocial support that people with MS require post-disclosure, in order to maintain their employment status. | Qualitative study using interviews. |
| AUS | Kirk-Brown, et al. (2014)^[20]^ | Physical: MS | N=1438 patients with MS | To statistically evaluate the relationship between disclosure of diagnosis at work and maintenance of employment. | Quantitative study using self-report surveys. |
| AUS | Netto et al. (2016)^[94]^ | Mental | N=12 people with mental illness | To generate a rich description of the meaning of employment for people with mental illness and identify the facilitators and barriers they experience in gaining and sustaining employment. | Qualitative study using focus groups and individual interviews. |
| AUS | Reavley et al. (2017)^[33]^ | Mental | N=5220 people with mental health problems | To carry out a national population-based survey in order to estimate the prevalence and explore the nature of experiences of work and education-related avoidance, discrimination and positive treatment in people with mental health problems. | Quantitative study using a survey involved computer-assisted telephone interviews. |
| AUS | Tynan et al. (2016)^[114]^ | Mental | N=1.457 employees from eight coalmine sites in Australia | To investigate the frequency of contact with professional and non-professional sources of support, and to determine the socio-demographic and workplace factors associated. | Quantitative study using a paper-based survey. |
| AUS | Vickers (2012)^[93]^ | Physical: MS | N=1 individual with MS | To examine one example of how workers with MS or any disability or stigmatising trait might experience working in teams. | Qualitative study: phenomenological case study |
| AUS | Werth (2015)^[40]^ | Mental and physical: chronic illness | N=24 working women with chronic illness | To understand attitudes of managers regarding chronic illness, because they play a significant role in the success of managing work and chronic illness. | Qualitative study, using interviews. |
| GBR | Brohan et al. (2014)^[95]^ | Mental | N=45 mental health service users | To provide a qualitative account of the beliefs and experiences of mental health service users regarding disclosure in employment contexts. | Qualitative study using interviews. |
| GBR | Coffey et al. (2014)^[29]^ | Physical: VI | N=120 visually impaired women | To explore barriers to employment for VI women and potential solutions to those barriers. | Mixed methods study using exploratory interviews, a survey and in-depth interviews. |
| GBR | Henderson et al. (2012)^[106]^ | Mental and physical | N=19 sick doctors from the UK | To explore the views of sick doctors on the obstacles preventing them returning to work. | Qualitative study using interviews |
| GBR | Jacoby et al. (2005)^[42]^ | Physical: epilepsy | N=560 employers | To re-examine employer attitudes to employment of people with epilepsy in the UK. | Quantitative study using surveys. |
| GBR | Marwaha et al. (2005)^[92]^ | Mental: schizophrenia or bipolar affective disorder | N=15 people with schizophrenia or bipolar affective disorder | To identify the opinions of a purposive sample of patients with psychosis on themes related to employment. | Qualitative study using interviews. |
| GBR | Secker et al. (2001)^[97]^ | Mental | N=156 service users | To identify service users' employment, training and education needs as they perceived them, and through doing so to raise the profile of vocational needs among mental health professionals. | Mixed methods, using surveys/interviews, followed by focus groups. |
| GBR | Thornicroft et al. (2009)^[61]^ | Mental: schizophrenia | N=732 persons with schizophrenia | To describe the nature, direction, and severity of anticipated and experienced discrimination reported by people with schizophrenia. | Quantitative study using a survey. |
| GBR | Yoshimura et al. (2018)^[103]^ | Mental | N=5942 mental health service users in England | To examine whether psychiatric diagnosis is associated with likelihoods of experienced and anticipated workplace discrimination and the concealment of psychiatric diagnoses. | Quantitative study using a survey. |
| DEU | Foitzek et al. (2018)^[13]^ | Mental and physical: chronic illness | N=487 people with chronic health conditions | To identify factors that have a negative or positive impact on the work lives of persons with chronic health conditions; to explore the needs of these persons to maintain a job or return to work and to compare these results with respect to these persons’ occupational status. | Qualitative using questionnaires that were analysed using qualitative methods. |
| DEU | Rüsch et al. (2017)^[66]^ | Mental | N=56 active-duty soldiers with mental illnesses who received psychiatric inpatient treatment | To explore views on (self-)labelling as 'mentally ill', experiences of discrimination and coping, risks and benefits of (non)-disclosure, service use, disclosure decisions and consequences of disclosing. | Qualitative study using focus groups. |
| DEU | Rüsch et al. (2018)^[117]^ | Mental | N=301 unemployed individuals with mental health problems | To examine the role of disclosure attitudes for employment over time. | Quantitative study using a survey. |
| NLD | Brouwers et al. (2016)^[99]^ | Mental: MDD | N=834 persons with MDD | To assess if people with MDD anticipate and experience discrimination when trying to find or keep paid employment. | Quantitative study using a survey. |
| NLD | Stutterheim et al. (2017)^[70]^ | Physical: HIV/AIDS | N=10 health care providers living with HIV | To explore motivations for disclosure and concealment, reactions to disclosures, the impact of reactions, and coping with negative reactions. | Qualitative study using interviews. |
| NLD | Wagener et al. (2014)^[75]^ | Physical: HIV/AIDS | N=18 people living with HIV | To identify the employment-related concerns of people with HIV and to formulate the key questions for a multidisciplinary guideline. | Qualitative study using focus groups and individual interviews. |
| BEL | Baert et al. (2016)^[46]^ | Mental: depression | N=288 trios of job applications of unemployed candidates were sent to 288 vacancies | To access hiring discrimination based on disclosed depression in a direct and causal way. | Qualitative study using an experimental design. |
| BEL | Degroote et al. (2014)^[72]^ | Physical: HIV/AIDS | N=54 people living with HIV | To explore experiences of PLHIV in the workplace, especially concerning disclosure and adherence to antiretroviral therapy. | Qualitative using questionnaires that were analysed using qualitative methods. |
| SUI | Corrigan et al. (2012)^[24]^ | Mental: SMI | N=85 persons with SMI | To examine the relationship of public stigma on current and lifetime histories of work. | Quantitative study using surveys. |
| SUI | Nebiker-Pedrotti et al. (2009)^[54]^ | Physical: diabetics | N=509 insulin-treated diabetics | To investigate the prevalence and risk factors of perceived diabetes-related discrimination in the workplace and in work-related insurances in persons with diabetes mellitus in Switzerland. | Quantitative study using surveys. |
| LTU | Endriulaitienė (2019)^[115]^ | Mental: job burnout | N=327 234 professionals (111 social workers and 123 psychologists) from Lithuania and 93 professionals (33 counsellors, 23 social workers, and 37 psychologists) from the USA | To explore the correlation between job burnout and self-stigma of seeking help among nonmedical mental health care providers (psychologists, social workers, and counsellors) in two countries – Lithuania and the US. | Quantitative design using a self-reported questionnaire. |
| ITA | Lasalvia et al. (2014)^[101]^ | Mental: psychosis | N=97 first-episode psychosis patients | To describe patterns of experienced and anticipated discrimination in a sample of persons experiencing first episode of psychosis and to explore associations with clinical and psychosocial variables. | Quantitative study using a survey. |
| ESP | Magallares et al. (2011)^[105]^ | Physical: Obesity | N = 110 obese patients | To examine the effect of work discrimination on the well-being of obese people. | Quantitative study using surveys. |
| POL | Obara-Gołębiowska (2016)^[77]^ | Physical: obesity | N=420 obese women | To analyse personal, subjective experiences related to weight bias and discrimination against obese people in the workplace of obese Polish women. | Qualitative study using group interviews. |
| RSA | Sprague et al. (2011)^[58]^ | Physical: HIV/AIDS | N=1.021 people who work for civil society organisations. N=1.086 people living with HIV | To present findings from three surveys of people living with HIV and civil society organisations about the experience of employment discrimination and stigma in the workplace. | Quantitative study using survey data from three sets of original data. |
| TUR | Öz et al. (2019)^[32]^ | Mental: schizophrenia | N=33 patients with schizophrenia (n = 25) and the human resource managers of selected companies (n = 8) | To identify the problems related to the placement of individuals with schizophrenia into jobs, the conditions allowing them to keep working and to reveal obstacles to their employment. | Qualitative study using interviews. |
| TUR | Üçok et al. (2012)^[102]^ | Mental: schizophrenia | N=732 persons with schizophrenia | To evaluate the level of anticipated discrimination in people with schizophrenia. | Quantitative study using a survey. |
| CHN | Liu et al. (2012)^[43]^ | Physical: HIV/AIDS | N=156 employers | To examine HIV-related stigma as a predictor of unemployment of people living with HIV/AIDS. | Quantitative study using surveys. |
| CHN | Rao et al. (2008)^[44]^ | Physical: HIV/AIDS | N=100 employers | To investigate employers' attitudes and hiring practices towards people with HIV across three culturally and linguistically distinct cities: Chicago, Beijing, and Hong Kong. | Qualitative study, using interviews. |
| CHN | Zhu et al. (2016)^[80]^ | Mental | N=1031 employees | To examine what aspects of knowledge, attitude, and previous contacts with people with mental illness influence working adults' intention to have future contact with people (e.g. to work with, to live nearby) with mental illness. | Quantitative study, using a cross-sectional telephone survey. |
| KOR | Park et al. (2010)^[71]^ | Physical: cancer | N=748 cancer patients | To provide a comprehensive overview of the relationship between changes in employment status and discrimination following a diagnosis of cancer. | Quantitative study using surveys. |
| KOR | Shim et al. (2016)^[81]^ | Physical: cancer | N=2000 cancer survivors returning to work | To investigate the attitudes of the public towards cancer survivors returning to work in Korea and to identify the factors influencing this negative attitude. | Quantitative study using a self-administered questionnaire. |
| VNM | Ishimaru et al. (2017)^[89]^ | Physical: HIV, HPB, HCV | N=400 nurses | To determine the factors associated with nurses’ attitudes towards accepting co-workers with HIV, HBV, or HCV. | Quantitative study using a self-administrated questionnaire. The study design was descriptive and cross-sectional. |
| VNM | Luu (2019)^[76]^ | Physical | N=502 employees with physical disabilities from companies based in Ho Chi Minh City, Vietnam | To examine how benevolent leadership contributes to the well-being of employees with disabilities. | Quantitative study using a survey. |
| JPN | Ishimaru et al. (2016)^[87]^ | Physical: HBV/HCV | N=992 nurses | To investigate barriers to nurses accepting the employment of HBV/HCV-infected colleagues within healthcare settings after appropriate risk assessment. | Quantitative study using online questionnaires. |
| HKG | Lee et al. (2005)^[53]^ | Mental: schizophrenia | N=480 out-patients with schizophrenia (n = 320) and diabetes (n = 160) | To document and compare the interpersonal experiences of stigma in patients with schizophrenia and patients with diabetes mellitus in Hong Kong. | Mixed method design using focus groups and a self-report questionnaire. |
| PAK | Noor et al. (2015)^[84]^ | Physical: HCV | N=228 employed HCV patients who had been admitted in Pakistani hospitals | To examine the impact of workplace bullying on self-esteem, including the mediating effect of internalized stigma and the moderating effect of spirituality, among HCV patients. | Quantitative study using questionnaires. |
| IND | Thomas et al. (2019)^[39]^ | Mental: SMI | N=13 patients with SMI | To understand the challenges faced by persons with SMI with professional degrees in obtaining and maintaining employment. | Qualitative study using interviews |
| NGA | Aguwa et al. (2016)^[85]^ | Physical: HIV/AIDS | N=489 people living with HIV/AIDS attending antiretroviral clinics | This study assessed the prevalence of workplace stigma and discrimination among people living with HIV/AIDS attending antiretroviral clinics in health institutions in Enugu, southeast Nigeria. | Quantitative study, a descriptive study was done, using a self-administered questionnaire. |
| NGA | Utuk, et al. (2017)^[90]^ | Physical: HIV/AIDS | N= 403 permanent and temporary employees in medium and large scale industries | To assess the level of stigmatising attitude towards co-workers living with HIV in the workplace. | Quantitative study using pre-tested semi-structured questionnaires. |
| KEN | Ebuenyi et al. (2019)^[100]^ | Mental | N=72 persons with mental disabilities | To report experienced and anticipated discrimination and social functioning in persons with mental disabilities in Kenia and to investigate the association between experienced and anticipated discrimination, social functioning, and employment in this population. | Quantitative study using surveys in a cross-sectional design. |
| GHA | Ulasi et al. (2008)^[88]^ | Physical: HIV/AIDS | N=104 adults from the four sub-districts in Kumasi | To assess HIV/AIDS-related stigma and discrimination of people living with HIV/AIDS in Kumasi, Ghana. | Quantitative study using surveys. |
| BRA | Garrido et al. (2007)^[68]^ | Physical: HIV/AIDS | N=17 men living with HIV | To analyse the effect of the stigmatization and discrimination process in the work environment on the routine healthcare and well-being of men living with HIV/AIDS. | Qualitative study using group interviews. |

**Abbreviations**

| HCV | hepatitis C virus |
| --- | --- |
| SMI | serious mental illnesses |
| HIV/AIDS | human immunodeficiency virus/acquired immunodeficiency syndrome |
| ADSD | adductor spasmodic dysphonia |
| MDD | major depressive disorder |
| wrMTBI | work-related mild traumatic brain injury |
| MS | multiple sclerosis |
| VI | visually impaired |
| SCI | spinal cord injury |
| SPMI | severe and persistent mental illness |
| CP | cerebral palsy |
| HPB | hepatitis B |
